# Supplementary material for: MetaRibo-Seq measures translation in microbiomes
Source: Nat Commun. 2020 Jun 29;11:3268. doi: 10.1038/s41467-020-17081-z (PMC7324362; doi:10.1038/s41467-020-17081-z)
Supplement: Supplementary file 10 — Supplementary Data 7 [file 41467_2020_17081_MOESM10_ESM.zip › File2/Confidence_High_Taxonomy/181989_out.krona.html]

Javascript must be enabled to view this page.

members
magnitude
magnitudeUnassigned
count
unassigned
taxon
rank

181989\_out

103

superkingdom
103
2

976
1
phylum

class
1
200643

order
1
171549

family
815
1

genus
1
816

species

SRS016335\_contig\_number\_13476
821
1

phylum
1239
102

class
88
186801

186802
88
2
order

SRS017821\_contig\_number\_contig-100\_11833.141119SRS024435\_contig\_number\_contig-100\_3776.39065

family
31979
1

1
1485
genus

species

SRS143466\_contig\_number\_25570
1
1262778

216572
family

SRS012273\_contig\_number\_27522SRS015579\_contig\_number\_5507SRS018836\_contig\_number\_19534SRS019968\_contig\_number\_contig-100\_14557.51882SRS022071\_contig\_number\_31774SRS022609\_contig\_number\_contig-100\_12186.105329SRS042628\_contig\_number\_contig-100\_13019.83902SRS048870\_contig\_number\_contig-100\_10007.87707SRS065504\_contig\_number\_contig-100\_14120.127613SRS077641\_contig\_number\_contig-100\_2257.43597SRS142890\_contig\_number\_contig-100\_16739.119040SRS892882\_contig\_number\_contig-100\_19331.19332
13
12

genus
1
459786


SRS143895\_contig\_number\_34558
species
1
1897011

541000
3
family

genus
1
292632

1897022
1

SRS144537\_contig\_number\_44037
species

genus

SRS063040\_contig\_number\_contig-100\_6849.185974
1
216851


SRS1041039\_contig\_number\_17181
species
1898205
1

68
172733

SRS011084\_contig\_number\_contig-100\_4678.183677SRS011302\_contig\_number\_21812SRS012969\_contig\_number\_26173SRS013098\_contig\_number\_22229SRS013476\_contig\_number\_contig-100\_12441.165588SRS013521\_contig\_number\_contig-100\_6906.67695SRS013965\_contig\_number\_30083SRS015431\_contig\_number\_23977SRS015663\_contig\_number\_3210SRS015694\_contig\_number\_20993SRS015960\_contig\_number\_6663SRS016018\_contig\_number\_14549SRS017307\_contig\_number\_6611SRS018351\_contig\_number\_5227SRS018427\_contig\_number\_13759SRS019068\_contig\_number\_106195SRS019496\_contig\_number\_946SRS019685\_contig\_number\_contig-100\_3478.92805SRS021948\_contig\_number\_contig-100\_10815.145038SRS023526\_contig\_number\_contig-100\_13761.164671SRS023914\_contig\_number\_20736SRS024132\_contig\_number\_contig-100\_12297.216873SRS045004\_contig\_number\_12333SRS045645\_contig\_number\_19170SRS045826\_contig\_number\_15297SRS047433\_contig\_number\_11003SRS049773\_contig\_number\_32314SRS049896\_contig\_number\_14359SRS050752\_contig\_number\_17160SRS053398\_contig\_number\_contig-100\_10676.114953SRS054352\_contig\_number\_22071SRS056273\_contig\_number\_17766SRS062654\_contig\_number\_contig-100\_29568.29569SRS063190\_contig\_number\_13530SRS064276\_contig\_number\_32395SRS064757\_contig\_number\_7723SRS075078\_contig\_number\_30372SRS075341\_contig\_number\_25439SRS076756\_contig\_number\_547SRS077024\_contig\_number\_14064SRS077194\_contig\_number\_18328SRS077392\_contig\_number\_36201SRS077849\_contig\_number\_4775SRS078242\_contig\_number\_11484SRS078419\_contig\_number\_33783SRS098644\_contig\_number\_40807SRS101376\_contig\_number\_contig-100\_3290.125301SRS1041031\_contig\_number\_23133SRS1041133\_contig\_number\_18946SRS1041139\_contig\_number\_3239SRS1041140\_contig\_number\_2211SRS1041145\_contig\_number\_37972SRS1041157\_contig\_number\_3322SRS104912\_contig\_number\_2476SRS142503\_contig\_number\_42328SRS144183\_contig\_number\_17752SRS144506\_contig\_number\_45077SRS144753\_contig\_number\_contig-100\_4010.4011SRS146812\_contig\_number\_contig-100\_10094.228971SRS147346\_contig\_number\_19617SRS147614\_contig\_number\_2420SRS148196\_contig\_number\_contig-100\_1866.194483SRS148253\_contig\_number\_11986SRS893172\_contig\_number\_contig-100\_3069.82672SRS893187\_contig\_number\_contig-100\_11819.82844SRS893373\_contig\_number\_16745SRS893383\_contig\_number\_36870SRS971275\_contig\_number\_5483
species

species

SRS143417\_contig\_number\_contig-100\_1807.118846
1898207
1

1263002
2

SRS063985\_contig\_number\_9684SRS147557\_contig\_number\_386
species

1263003
12

SRS011239\_contig\_number\_contig-100\_2146.168159SRS020869\_contig\_number\_16922SRS021484\_contig\_number\_9052SRS049959\_contig\_number\_48602SRS049995\_contig\_number\_19334SRS075773\_contig\_number\_24504SRS075821\_contig\_number\_contig-100\_1101.51319SRS077231\_contig\_number\_16296SRS078176\_contig\_number\_20626SRS140513\_contig\_number\_12074SRS143523\_contig\_number\_contig-100\_7468.7469SRS893279\_contig\_number\_14678
species
